# Adaptable mesocosm facility to study oil spill impacts on corals

**DOI:** 10.1002/ece3.5095

**Published:** 2019-04-17

**Authors:** Denise P. Silva, Gustavo Duarte, Helena D.M. Villela, Henrique F. Santos, Phillipe M. Rosado, João Gabriel Rosado, Alexandre S. Rosado, Edir M. Ferreira, Adriana U. Soriano, Raquel S. Peixoto

**Affiliations:** ^1^ LEMM, Laboratory of Molecular Microbial Ecology, Institute of Microbiology Paulo de Góes Federal University of Rio de Janeiro (UFRJ) Rio de Janeiro Brazil; ^2^ IMAM‐AquaRio – Rio de Janeiro Aquarium Research Center Rio de Janeiro Brazil; ^3^ Biotechnology Section, Leopoldo Américo Miguez de Mello Research & Development Center – CENPES PETROBRAS Rio de Janeiro Brazil; ^4^ Present address: Department of Marine Biology Fluminense Federal University Rio de Janeiro Brazil

**Keywords:** coral reef, flow‐through system, marine mesocosm, marine pollution, *Millepora alcicornis*, oil spill

## Abstract

Although numerous studies have been carried out on the impacts of oil spills on coral physiology, most have relied on laboratory assays. This scarcity is partly explained by the difficulty of reproducing realistic conditions in a laboratory setting or of performing experiments with toxic compounds in the field. Mesocosm systems provide the opportunity to carry out such studies with safe handling of contaminants while reproducing natural conditions required by living organisms. The mesocosm design is crucial and can lead to the development of innovative technologies to mitigate environmental impacts. Therefore, this study aimed to develop a mesocosm system for studies simulating oil spills with several key advantages, including true replication and the use of gravity to control flow‐through that reduces reliance on pumps that can clog thereby decreasing errors and costs. This adaptable system can be configured to (a) have continuous flow‐through; (b) operate as an open or closed system; (c) be fed by gravity; (d) have separate mesocosm sections that can be used for individual and simultaneous experiments; and (e) simulate the migration of oil from ocean oil spills to the nearby reefs. The mesocosm performance was assessed with two experiments using the hydrocoral *Millepora alcicornis* and different configurations to simulate two magnitudes of oil spills. With few exceptions, physical and chemical parameters remained stable within replicates and within treatments throughout the experiments. Physical and chemical parameters that expressed change during the experiment were still within the range of natural conditions observed in Brazilian marine environments. The photosynthetic potential (*F_v_/F_m_*) of the algae associated with *M. alcicornis* decreased in response to an 1% crude‐oil contamination, suggesting a successful delivery of the toxic contaminant to the targeted replicates. This mesocosm is customizable and adjustable for several types of experiments and proved to be effective for studies of oil spills.

## 1 INTRODUCTION

Coral reefs are sensitive biological communities with high biodiversity and productivity (Ainsworth, Thurber, & Gates, 2010; Richmond, 1993) that play a critical role in the trophic interactions and connectivity of marine ecosystems (Säwström et al., 2016). They also have high economic value for local communities, for instance through fishing and tourism (Spalding et al., 2017). Despite their ecological and economical importance, coral reefs are experiencing serious declines and impacts caused by different factors, including anthropogenic stressors, such climate change, sedimentation, and pollution (Hughes et al., 2018; Leite et al., 2018; Liu, Meng, Liu, Wang, & Leu, 2012; Nepote, Bianchi, Chiantore, Morri, & Montefalcone, 2016; Nyström, Folke, & Moberg, 2000; Reichelt‐Brushett & Harrison, 1999). These disturbances can, independently or synergistically, induce coral mortality, contribute to disease outbreaks, and affect coral reproduction and recruitment (Muthukrishnan & Fong, 2014; Richmond, 1993; Richmond, Tisthammer, & Spies, 2018).

Oil spills have been affecting reef ecosystems for decades. In view of the high demand for petroleum products, there is a need to increase crude‐oil extraction, and accidental leaks can occur throughout the production chain from offshore platforms, distribution pipelines, and tankers. Corals can be impacted by these spills due to the rapid incorporation of the water‐soluble fraction (WSF) of oil (NOAA, 2010; Turner & Renegar, 2017). Sublethal effects of oil contamination on corals can also include decreased growth rates (Guzmán & Holst, 1993; Guzmán, Jackson, & Weil, 1991; Prouty, Fisher, Demopoulos, & Druffel, 2014; Xu et al., 2018); the accumulation of polycyclic aromatic hydrocarbons (PAHs) in coral tissue, which can cause bleaching (Harrison, Collins, & Alexander, 1990; Ko, Chang, & Cheng, 2014); excessive mucus production (Hsing et al., 2013); shifts in the composition of the coral microbiome (Santos et al., 2015); and decreases in settlement and development of coral larvae (Epstein, Bak, & Rinkevich, 2000; Loya & Rinkevich, 1979). DeLeo, Ruiz‐Ramos, Baums, and Cordes (2015) demonstrated the toxic effects of oil and dispersants—a chemical remediation strategy largely applied in oil spills—on three deep‐water coral species: *Paramuricea* type B3, *Callogorgia delta*, and *Leiopathes glaberrima*. Ruiz‐Ramos, Fisher, and Baums (2017) also showed that oil that had been previously dispersed by chemical remediation was harmful to black coral *L. glaberrima* as evidenced by enhanced expression of coral microbiome genes associated with stress. Santos et al. (2015) also demonstrated the negative effect of oil on the photosynthetic capacity of the coral‐associated symbiotic algae, often used as an indirect proxy for coral health. Field studies provide the best opportunity to simulate complex dynamics of contaminants within environments to understand the effects of oil spills under realistic conditions (Culp & Baird, 2006; NOAA, 2010) and to test mitigation strategies. However, the number of uncontrolled variables in field studies and eventual lack of reliable control areas can make it difficult to disentangle the consequences of oil pollution from other environmental factors in marine environments (Adams, 2003). In addition, field experiments where toxic contaminants are released into the environment must be avoided when possible or extremely well contained.

Micro and mesocosms, which are structures of various sizes (capacity 1 L to >10,000 L), can be used to understand the effects of different stressors on corals (Petersen, Cornwell, & Kemp, 1999; Putnam, Barott, Ainsworth, & Gates, 2017; Reilly, 1999). Field conditions can be reproduced by mesocosms that use specially designed equipment to control and manipulate chemical and physical parameters that mimic natural environmental conditions (Duarte et al., 2016; Luckett, Adey, Morrissey, & Spoon, 1996; Odum, 1984). For a mesocosm system to be considered acceptable, it must provide similar conditions to the natural environment, true replication over time, self‐sustaining conditions, and the establishment of a representative biological community (Alexandre, Luiker, Finley & Culp, 2016; Riebesell, Fabry, Hansson, & Gattuso, 2010). The advantages of using mesocosms include better reproduction of environmental conditions compared to laboratory bioassays and high‐quality, reproducible data which are easier to collect and interpret than in conventional field studies. For instance, Duarte et al. (2016) designed a mesocosm to study the impacts of heat stress and acidification on corals. The authors found that the mesocosm system allowed the measurement of ecological, biological, and physiological stress responses of corals to a variety of environmental parameters. In addition, mesocosms are very versatile and can be adapted to work with a variety of stressors. However, there are some limitations, such as the use of water pumps, which can fail, and the existence of some degree of pseudoreplication due to drawing water from a common stock tank.

Here, we describe a new, realistic mesocosm system that is low cost yet can be effectively controlled to understand the effects of oil spills on corals. This system can also be used to further develop and test potential remediation strategies, taking into consideration the importance of maximizing the reproduction of natural conditions and the use of true replicates. In addition, we demonstrate how this system improves the known limitations of previously described mesocosms, such as the dependency of pumps to control flow, thereby reducing costs and avoiding clogs. This system can be adapted for other experiments with or without oil, among others. Finally, we evaluated the efficacy of the mesocosm design by conducting two experiments with different configurations: (a) a continuous flow‐through and open system, fed by gravity with seawater recirculation in storage tanks and dilution of contaminants, and (b) a continuous flow‐through and open system, fed by gravity using seawater from contaminant tanks without recirculation in storage tanks and without dilution of contaminants. These configurations allowed us to simulate two different situations of coral exposure to a crude‐oil spill with (a) diluted or low‐magnitude contamination and (b) with direct exposure to a large and acute oil spill.

## 2 MATERIALS AND METHODS

### 2.1 Ethics approval and consent to participate

Permission to collect *Millepora alcicornis* was obtained from the Municipal Secretary of the Environment and Fish of Armação dos Búzios, license numbers 0093/2014 and 007/2015. The microbial survey permit was obtained from CNPq (National Council for Scientific and Technological Development) and SisGen number A620FE5.

### 2.2 PROCORAIS Mesocosm

**Figure 1:**
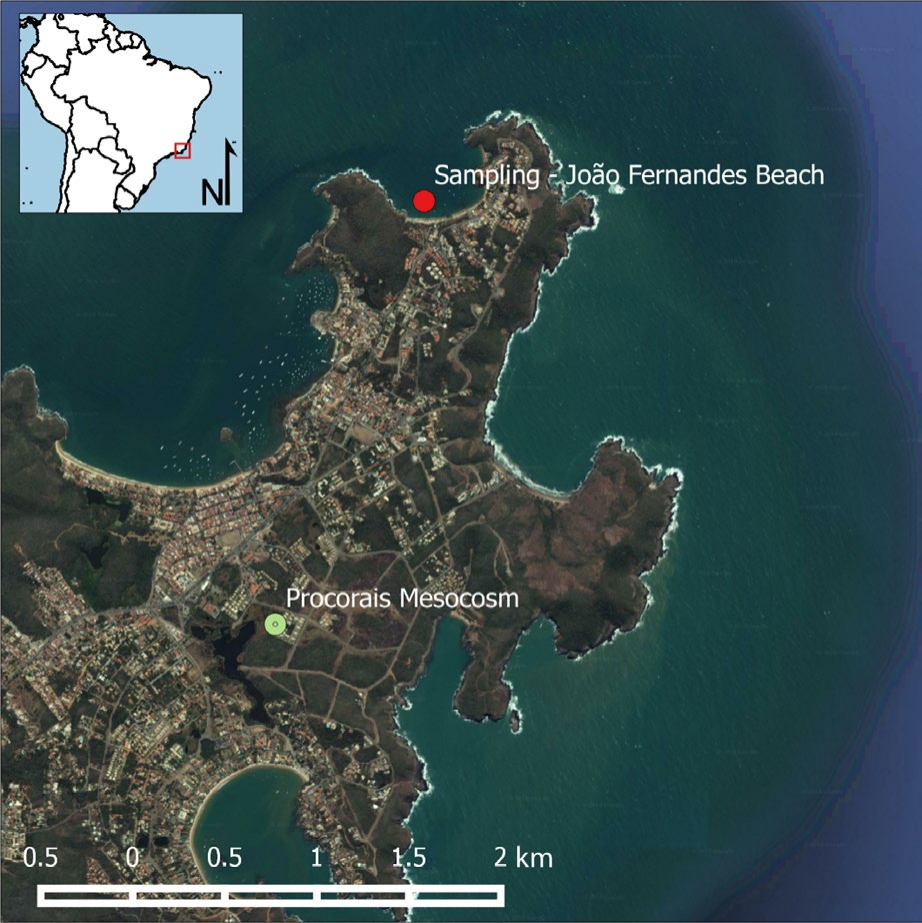
Map of the location of João Fernandes, Beach (22°44′29.95″; 41°52′35.62″W), and the PROCORAIS mesocosm (22°45′44.22″S; 41°53′3.97″W), Armação dos Búzios, Rio de Janeiro

The PROCORAIS Mesocosm was located at the Center for the Study of Oil Bioremediation in Marine Environments, a joint initiative of Federal University of Rio de Janeiro (UFRJ) and Petrobras Research & Development Center (CENPES), in the city of Armação dos Búzios (22°45′44.22″S; 41°53′3.97″W), state of Rio de Janeiro, Brazil (Figure 1). Natural sunlight was chosen to reproduce the light spectrum experienced by corals. The location and best position to replicate natural light conditions within the structure were selected using SketchUp Pro software (Trimble Navigation Limited, 2015), which made it possible to analyze the profiles of shadows of nearby buildings and variations in luminosity throughout the year in this area.

**Figure 2:**
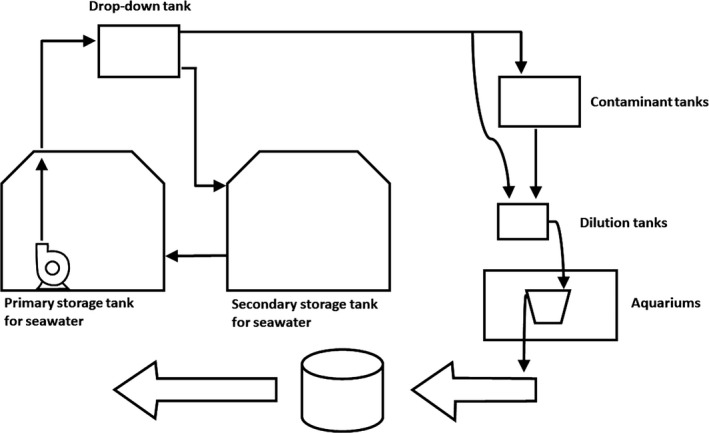
Schematic flowchart of seawater flow in the mesocosm system. Seawater is stored in the primary and secondary tanks and pumped to the drop‐down tank. By gravity, the water flows from the drop‐down tank and is distributed to the contaminant tanks. The dilution tanks receive the treatments and by gravity supply the aquariums with corals. The continuous experimental waste flow is collected by a single pipe, which feeds into a wastewater treatment system

The mesocosm can be configured to (a) have continuous flow‐through, (b) operate as an open or closed system, (c) be fed by gravity, avoiding pump clogging, (d) have independent mesocosm sections that can be used separately in simultaneous experiments, and (e) simulate the migration of oil from ocean oil spills to nearby reefs. The mesocosm can hold up to 13 treatments (52 independent stock tanks and 52 independent dilution tanks in total) with four replicates each, with the option to reconfigure the system for fewer treatments with greater replication. This mesocosm system is partitioned into three sections: (a) seawater storage, (b) contaminant stock (representing the open ocean where the oil spill and weathering would occur), and (c) experimental aquariums (representing the “reefs”). A schematic flowchart of the mesocosm system is shown in Figure 2. The path from the tanks to the aquariums represents the path of contaminated water from the spill area to the reef.

#### 2.2.1 Seawater storage section

**Figure 3:**
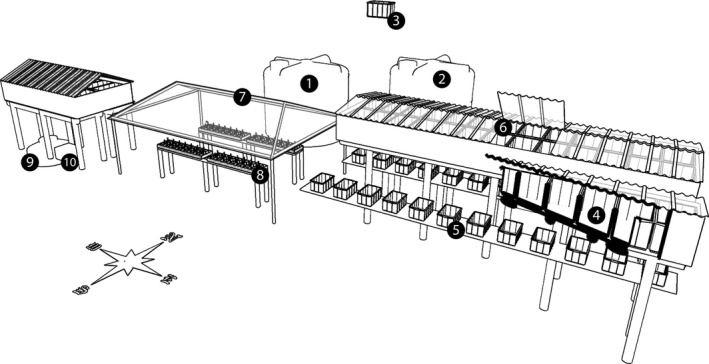
Plan of the mesocosm system: *Seawater storage section*: primary seawater storage tank (1), secondary seawater storage tanks (2), drop‐down tank (3); *Contaminant stock section: Contaminants tanks*(4) and (6) dilution tanks (5); *Experimental aquariums section*:polycarbonate roof (7); aquariums and water baths (8); cold‐water reservoir (9) and Chiller unit (10)

Seawater flows continuously between two 20,000‐L polyethylene storage tanks (Fortlev^®^, Camaçari, Bahia, Brazil) at ground level and a 300‐L drop‐down polypropylene tank (Moar Plasticos^®^, São Paulo, São Paulo, Brazil) 6 m above the ground (Figures 2 and 3). From the first storage tank, seawater is drawn to the second 20,000‐L polyethylene storage tank through a 60‐mm‐diameter overflow pipe (Tigre^®^, Joinville, Santa Catarina, Brazil) which gravity‐feeds seawater to the contaminant tanks through 4 PVC pipes (Figures 2 and 3). Excess seawater from the drop‐down tank is recirculated to the two 20,000‐L tanks to ensure oxygenation and homogeneous conditions within the entire storage and distribution system. The system uses a single 20,000 L/hr water‐lift pump (JEBO Lifetech Sp620, Monterey Park, California, USA) in the seawater storage section. By distributing fluids through gravity feed, the system avoids clogging, a common problem in experimental work with hydrocarbons. In addition, the use of a single pump reduces implementation and maintenance costs, factors that can be constraining in mesocosm systems. Finally, the gravity‐feed distribution tank provides a fail‐safe for the system because seawater can continue to flow if the single pump fails and a replacement must be installed.

#### 2.2.2 Stock of contaminants

The section housing contaminant stocks (Figure 2) was composed of up to 52 (maximum capacity of the mesocosm) 300‐L polypropylene contaminants tanks (Moar Plásticos^®^, Campinas, São Paulo). These tanks were housed on three platforms 3 m above the ground. Each stock tank was paired with a 26‐L polypropylene dilution tank (Moar Plásticos^®^, Campinas, São Paulo) that was also gravity‐fed and had an air bubbler to better homogenize the treatments. The 26‐L dilution tanks were also connected directly to a 300‐L drop‐down tank containing pure seawater. Thus, the contaminant concentration could be adjusted precisely by controlling the flow from the distribution and stock tanks to the dilution tanks. Water levels in all stock and dilution tanks were maintained with a float switch to simulate the gradual release of the WSF fraction of the oil into the water column, according to dilution observed in marine systems, rendering the oil concentration lower over time. All dilution tanks had an air bubbler to homogenize the dilution. All tanks were maintained at ambient air temperature.

The dilution tanks also had an overflow connected to a short section of 6.5‐mm polyethylene hose (internal diameter; Rubberplastic, Cajamar, São Paulo) that was in turn connected to a 4.35‐mm polyethylene hose (internal diameter; Rubberplastic, Cajamar, São Paulo). This hose supplied contaminated seawater to the experimental aquarium section (Figure 2) that was connected to a last hose that fed the aquariums and controlled the entire flow of the system. This last hose was made of silicone (1.02 internal and 2.16 external diameter; Elastim, São Paulo) and provided a precise flow of 4.8 L/hr. The natural‐seawater intake system had a flow valve (3 × 3 × 3.5 cm; Mr. PET, Ipiranga, São Paulo) to allow the dilution to be adjusted by setting the compound concentration in the contaminant tanks as well as the incoming saltwater‐flow dilution tanks.

#### 2.2.3 Experimental aquariums section

**Figure 4:**
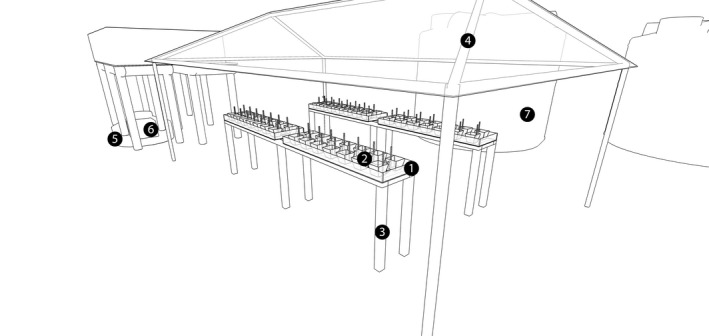
Schematic view of the experimental aquarium section (1), experimental aquariums (2); water‐bath stands (3); polycarbonate roof (4); cold‐water reservoir (5); chiller unit (6); two 20,000‐L stock‐water reservoirs (7)

The experimental aquariums section (Figures 2 and 4) of the mesocosm received seawater and the seawater–oil mixture from the dilution tanks. This section was composed of four water baths, each with an independent temperature control. In each water bath, up to 13 aquariums could be arranged, for a total of 52 aquariums. Each aquarium contained a small platform to facilitate sample and data collection with analytical instruments (e.g., the probes for measuring physical and chemical parameters). Each aquarium also had an individual air bubbler to mix the water and remove excess coral mucus that is produced in response to the contaminant (Mitchell & Chet, 1975), reducing the boundary‐layer effect. The mesocosm structure, including aquariums, was covered with a polycarbonate roof, and the luminosity was regulated with a 70% shade cloth (30% transmitted light; Equipesca, Campinas, São Paulo). The intensity of light that the aquariums received was 106.45 ± 1.95 µmol photons m^−2^ s^−1^ and was measured with an LI‐250 light meter with an LI‐190SA sensor (Li‐Cor, Lincoln, Nebraska, USA).

Each aquarium had an overflow that allowed the water received from the dilution tank to flow to a single wastewater treatment system. This wastewater treatment system was composed of an oil and water separator (ZP5000, Zeppini, São Bernardo do Campo, São Paulo) connected to an activated carbon filter (BOYU, Guangdong, China) for dissolved hydrocarbon retention. All water in the PROCORAIS Mesocosm system (including controls) was recovered, treated, and postdisposed in the conventional sewage system. Oil residues were stored and sent to the nearest point of collection and treatment. The wastewater treatment system had the capacity to receive 800 L/hr of water and 5 L/hr of oil which complied with the requirements of ABNT 14605‐2 NBR standards (ABNT, 2010) and the Federative Brazilian Regulation, CONAMA 430 (CONAMA, 2011).

Aquariums were immersed in the water bath and used a two‐part system to control and maintain temperatures (MT – 518Ri; Full Gauge Controls, Canoas, Rio Grande do Sul, Brazil). The temperature‐control sensor was placed in one randomly selected experimental aquarium in each of the four water baths. For water cooling, the mesocosm was connected to a 1,000‐L tank that was partly buried to take advantage of the thermal soil capacity to maintain water temperature. This stock tank was filled with 800 L of fresh water and connected to a water chiller with a titanium coil tube (Chiller 1/4 HP; Gelaqua, Santos, São Paulo) to maintain the fresh water reservoir at 18°C. This stock tank was connected to a 2000 L/hr pump (SB‐2000; Sarlo Better, São Paulo, São Paulo) that sent cold water to the water baths when the temperature controller was triggered.

### 2.3 Mesocosm validation

To validate the PROCORAIS Mesocosm for studying the effect of crude‐oil contamination on corals, two trials were performed with different configurations of the system. In both trials, our aim was to verify that the system could emulate realistic environmental conditions while maintaining consistent control on abiotic factors within replicates of differing treatments. The trials were conducted in April 2016 and April 2017 and were both run as open continuous flow‐through systems. During the interval between the experiments, all tanks and aquariums were cleaned and all 4.35‐mm‐ and 6.5‐mm‐diameter hoses were replaced. Both experiments used Marlim crude oil from the Campos Basin (Rio de Janeiro, Brazil). Seawater (CTRL) and crude oil (O) treatments were applied in both configurations (1 and 2), in quadruplicate as follows.

#### 2.3.1 Trial 1 (April, 2016)

In trial 1, our aim was to test the ability of the mesocosm for detecting impacts of the WSF of oil on the hydrocoral *M. alcicornis* under conditions that emulate an oil spill being naturally diluted by water currents. This trial was designed to simulate gradual release of oil to the water column at a dilution observed in natural systems (ocean water) reproducing a pulse of contaminated water to the reef that would slowly become less contaminated after some time. To achieve these conditions, the mesocosm was configured with continuous flow‐through as an open‐system experiment fed by gravity in the entire mesocosm. The gravity‐fed water flow was controlled by the small tanks between the 300‐L tanks and the aquariums. These intermediate tanks had a controlled water level that allowed the weight of the water column to be constant. The seawater was stored in the 20,000‐L tanks (seawater storage section) and the 300‐L tanks were refilled constantly in the controls and treatments. Marlim crude oil was added to the oil‐treatment tanks, which were diluted 10 times in the dilution tanks to reach a final concentration of 0.07% (v/v). The experiment was run for 30 days, with 9 days to acclimate the corals in the mesocosm and 21 days for the experiment. The physical, chemical, and biological parameters were measured every 2 days in the beginning and every 3 days after day 12 of experiment.

#### 2.3.2 Trial 2 (April, 2017)

In the second trial, our aim was to test the ability of the system to deliver a constant pollutant concentration to the aquariums throughout the experiment to test the effects of acute and constant oil contamination on corals. In this configuration, the following adjustments were carried out relative to trial 1. First, a higher concentration of Marlim crude oil (1%; v/v) was added to a final volume of 250 L of seawater in the contaminant tanks. Second, the treatments were not diluted in this trial, in order to simulate a high‐magnitude spill and increase the concentration of soluble hydrocarbons in the water. Instead, dilution tanks were used only to maintain the volume of water and allow the flow, by gravity, to be constant, even with the volume of the contaminant tanks decreasing over time. Third, treatments were circulated to the aquariums with a reduced flow through the 26‐L tanks, which allowed a flow rate of 0.72 L/hr, to enhance the exposure of the organisms to the WSF of the oil. In this trial, the seawater storage sector was not used, since dilution was not applied and seawater from the treatment tanks was enough to perform all the trial. This second experiment was run for 21 days, with 7 days for acclimating the corals in the mesocosm and 14 days for the experiment. The parameters were measured four times during the experiment, including the acclimation period.

#### 2.3.3 Collecting corals in the field

*Millepora alcicornis* colonies were collected for use in the experimental mesocosms from João Fernandes Beach, Armação dos Búzios (22°44′29.95″S; 41°52′35.62″W). During transportation, the nubbins were kept submerged in continuously renewed seawater under shade cloth with a capacity to block 70% of solar incidence and at the same water temperature as the collection site. *M. alcicornis* was chosen for this study because it is one of the most abundant coral species at Armação dos Búzios.

At the PROCORAIS mesocosm, the colonies of *M. alcicornis* were fragmented into pieces approximately 5 cm long. The coral was handled only by the tips to avoid obstructing and possibly damaging the polyps. The 5‐cm nubbins were placed in each experimental aquarium (1.2 L).

#### 2.3.4 Physical and chemical validation

##### Temperature, salinity, and pH validation

Temperature, salinity, and pH were measured during both trials of the PROCORAIS Mesocosm with a multiparameter probe (model HI 9,828; Hanna® Instruments, Tamboré, Barueri, São Paulo). We recorded these parameters every 2 days in the beginning of the experiment and every 3 days in trial 1 (eight measurements in total). In trial 2, we recorded these parameters four times (Days 0, 1, 4, and 13). In both experiments, we included the acclimation period (i.e., Day 0).

##### Analysis of nitrogen compounds

Seawater samples were collected from aquariums and filtered on cellulose membranes (0.45‐µm pore size and 47 mm diameter) for analyses of nitrogen compounds (nitrite, nitrate, and ammonium) in both trials. These samples were stored in amber bottles at −20°C, and the analyses were performed by the Laboratory of Environmental Analyses, Institute of Biology, UFRJ, through the FIAstar® 5,000—Application Note: 5,200 for nitrite, 5,202 for nitrate, and 5,220 for ammonium (Foss Analytical, Höganäs, Sweden) by flow injection process.

##### Dissolved organic carbon analysis

Dissolved organic carbon (DOC) was evaluated in both trials. Aquarium seawater samples were collected (60 mL) from each aquarium and filtered in 0.45‐µm pore size cellulose membranes. The membranes were stored in glass vials with Teflon caps with an addition of 50 µL phosphoric acid. The vials were stored at room temperature.

Samples were analyzed by the Multiuser Unit of Environmental Analysis, Institute of Biology, UFRJ, through the process of oxidation with sodium persulfate in a titanium furnace under high temperature and pressure. A Sievers InnovOx Total Organic Carbon (TOC) Analyzer was used to perform the analyses.

##### Quantification of petroleum hydrocarbons

To analyze total petroleum hydrocarbons (TPH) and polycyclic aromatic hydrocarbons (PAH) in both experiments, we collected 1 L of seawater from each replicate treatment aquarium (oil and control, four replicates each) in sterile amber glass bottles with Teflon caps. In trial 1, samples were taken at day 2 of experiment (36 hr after the oil was added in the contaminant tanks) day 5 and day 21 of experiment. In trial 2, samples were taken at day 2 of the experiment (36 hr after the oil was added in the mesocosm system), as well as days 4 and 14 of the experiment. All samples were stored at 4°C until analysis at about 24 hr after collection.

Hydrocarbons were extracted according to method 3510C (U.S. EPA, 1996). TPH analyses were performed using the gas chromatography technique with flame ionization detection (GC/FID) according to method 8015B (U.S. EPA, 2003). The 37 PAHs were detected by gas chromatography/mass spectrometry (GC/MS) using the 8270D method (U.S. EPA, 2007).

#### 2.3.5 Biological validation – Maximum photosynthetic capacity of photosystem II (*F_v_/F_m_*)

The efficacy of the mesocosm to detect biological effects of oil spills on corals was evaluated by comparing values of maximum photosynthetic quantum yield (*F_v_*/*F_m_*) among treatments and controls. *F_v_*/*F_m_* serves as an estimate of the associated‐photosynthetic algae health and can directly infer the physiological state of corals in the treatments. The maximum quantum yield of photosystem II was determined from the fluorescence values, termed *F_o_* and *F_m_*. While basal or minimal fluorescence (*F_o_*) corresponds to the signal emitted under nonactinic modulated illumination (~1.5 μmol photons m^−2^ s^−1^), maximum fluorescence (*F_m_*) was obtained by exposure to a pulse of saturating light (6,000 μmol photons m^−2^ s^−1^) in the presence of modulated light. The difference between these extreme values corresponds to the variable fluorescence value (*F_v_*) and the *F_v_/F_m_* ratio represents the maximum quantum efficiency of photosystem II. Measurements of *F_v_*/*F_m_* were taken at the same time and frequency as the physical and chemical parameters with a subaquatic pulse and amplitude‐modulated fluorometer (Diving‐PAM; Heinz Walz, Effeltrich, Germany) with the following configuration: measuring light intensity (MI) = 6; saturation pulse intensity (SI) = 8; saturation pulse width (SW) = 0.8; gain (G) = 1; damping (D) = 1. To avoid interference from diurnal photo‐inhibition artifacts, measurements were taken 1 hr after sunset to ensure full recovery of the reaction centers. The intensity of the photosynthetically active radiation in each aquarium was measured using a Fiber Quantum Sensor (1 mm diameter) connected to the Diving‐PAM. We positioned the probe on the tissue of coral polyps, using one randomly selected polyp per aquarium, and the same coral nubbin (from each replicate, *n* = 4 nubbins) was used to measure chlorophyll fluorescence at different sampling times. The hydrocorals were acclimated in the dark for 20 min, as this dark period results in the disappearance of the nonphotochemical processes from the dissipation of the photosystem II excitation energy.

#### 2.3.6 Statistical analysis

Statistical comparisons of the repeated measurements were performed with the nlme package (Pinheiro, Bates, DebRoy, & Sarkar, 2017) in the R statistical software (R Core Team, 2017). For this analysis, we used the lme function (Pinheiro et al., 2017) to create a mixed effects model with Day and Treatment (control or oil) as interactive fixed effects and aquarium identity as a random effect. We then conducted a post hoc analysis to estimate the least square means and to identify statistically significant (*p* < 0.05) pairwise interactions between Day and Treatment (lsmeans package; Lenth, 2016) with Tukey tests. We log‐transformed the physio‐chemical measurements as needed to meet the assumptions of normality and homoscedasticity of model residuals.

## 3 RESULTS

### 3.1 Mesocosm validation

#### 3.1.1 Chemical and physical parameters

##### pH, temperature, and salinity

**Figure 5:**
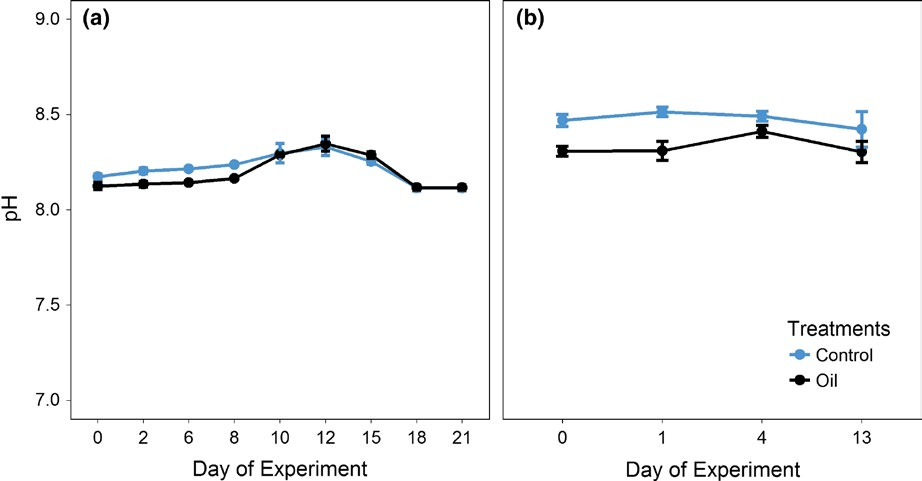
Measurements of pH during trial 1 (a) (21 days) and trial 2 (b) (14 days) in the treatments: CTRL (Control) and O (Oil)

**Figure 6:**
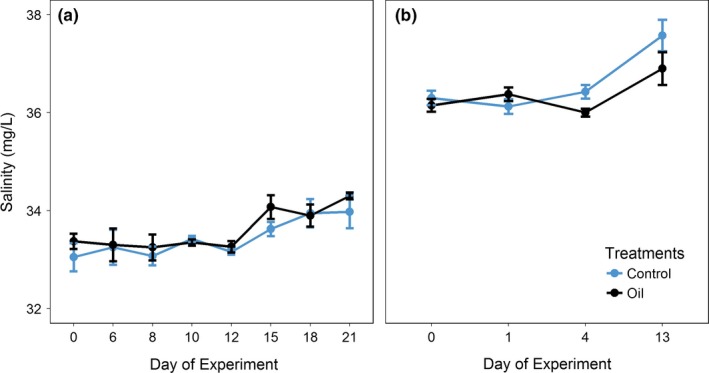
Measurements of salinity during trial 1 (a) (21 days) and trial 2 (b) (14 days) in the treatments: CTRL (Control) and O (Oil)

In trial 1, (Figure 5a) a significant difference of pH values was observed throughout time (*p* < 0.0001). In the both trials, the pH measurements increased at the same time. A significant difference in pH values was also observed between treatments (*p* = 0.045) for trial 2, but not over time (Figure 5b). Temperature was controlled by a water bath and monitored throughout the experiments and remained stable at 24°C in all aquariums, and no statistical differences were detected among treatments or across time. In both trials 1 and 2, salinity did not differ significantly between the treatments (*p* > 0.05; Figure 6a,b), but both trials were significantly different over time (*p* < 0.001 for trial 1 and *p* < 0.003 for trial 2), with an increase in salinity on the last days of each experiment compared with the first day.

##### Analysis of nitrogen compounds

Nitrate, ammonium, and nitrite measurements were below the detection limit in the first trial (6.8 µ/L, 5.9 µ/L, and 1.0 µ/L, respectively). In the second trial, nitrate levels did not differ significantly between treatments throughout time. Mean values of 58 ± 10 µ/L and 43 ± 20 µ/L were observed in the control and oil treatments, respectively. The nitrite levels were below the detection limit (0.9 µ/L). Ammonium levels did not differ as a function of the treatments (*p* = 0.39), but did differ over time (*p* = 0.01), with higher levels on day 4 compared with the first day.

##### DOC

In trial 1, DOC levels remained below the detection limit (0.004 mg/L) for all samples, while no difference (*p* = 0.413) was observed during trial 2 in DOC levels between treatments throughout time.

##### Detection of petroleum hydrocarbons

In the first trial, where oil contamination of 0.07% (v/v) was simulated, TPH and PAH fractions were below the detection limit (0.03 μg/L) in all treatments throughout the experiment. In the second trial, where a 1% (v/v) oil contamination was applied, TPH values differed significantly between treatments throughout time (*p* < 0.001). Least‐squared means showed that TPH in the oil treatment at day 13 was significantly higher when compared to oil treatment at day 2 and 4 (both with *p* < 0.001) and control at day 13 (*p* = 0.0070). Mean TPH throughout the experiment was 21.75 ± 14.90 μg/L for control and 67.41 ± 29.26 μg/L for the oil‐contaminated samples. PAH values of 0.90 ± 0.35 μg/L and 2.08 ± 0.5 μg/L were observed for control and oil‐contaminated samples, respectively. In trial 2, PAH was significantly higher in the oil treatment on the second day of the experiment when compared with all other days and treatment combinations (*p* = 0.01). However, PAH levels stabilized in the oil treatment by day 4 and were not different from other day–treatment combinations throughout the remainder of the experiment.

##### Maximum photosynthetic capacity of photosystem II (*F_v_*/*F_m_*)

**Figure 7:**
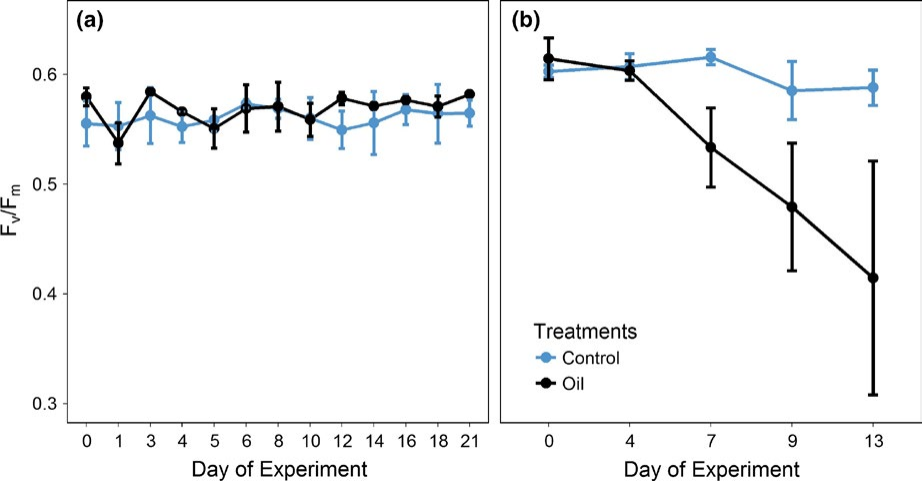
Measurements of photosynthetic quantum yield (*F_v_/F_m_*) of the algae associated with the hydrocoral *Millepora alcicornis* during trial 1 (a) (21 days) and trial 2 (b) (14 days) in the treatments: Ctrl (Control) and O (Oil)

For both trials, *F_v_*/*F_m_* values of the *M. alcicornis* symbiotic algae ranged between 0.55 and 0.65 during the acclimatization period; *F_v_/F_m_* above 0.5 indicates that the corals were well adapted to the mesocosm (Fitt, Brown, Warner, & Dunne, 2001). In trial 1, no significant differences were observed in *F_v_/F_m_* values between treatments. The mean values (0.566 ± 0.05 for the control and 0.567 ± 0.006 for the oil) were similar between replicates (Figure 7a) and overtime (*p* < 0.05). These results suggest that the photosynthetic potential of the associated symbiotic algae was not negatively affected by oil, due to its low concentration (0.07%), as well as the high degree of weathering, low solubility, and availability in the water. In the second trial, the *F_v_/F_m_* values obtained from oil‐contaminated aquariums decreased significantly from the day 4 (with a mean of 0.603 ± 0.009) until the end of the experiment (0.414 ± 0.106; Figure 7b).

## 4 DISCUSSION

The PROCORAIS Mesocosm was designed to perform experiments on better understanding the effects of oil spills on corals, with a large set of true replicates and treatments. The mesocosm was designed principally to be used as a continuous‐flow open system, and the materials chosen for its construction were carefully selected to not interact with the oil. This mesocosm can be easily adapted to test other environmental conditions, other contaminants, or with different marine organisms. In addition, the magnitude of contaminants is adjustable to test a range of scenarios. For example, the oil concentrations used in the validation experiments (0.07% and 1%) were chosen to simulate both low‐ and high‐magnitude oil spills, respectively. The application of 1% oil concentration was especially important to test the ability of the system to deal with this contamination level that can be determinant for the magnitude of a spill impact on coral reefs—that is, the system dealt with high concentrations of oil without clogging, being able to emulate a high‐magnitude spill, which is important to study the potential effects of a worst‐case scenario. Furthermore, the hydrocorals were held in isolated tanks, avoiding any exchange of microbiota, mucus, and/or products of coral metabolism among replicates or distinct treatments. The design minimizes the possibility of the spread of coral diseases, mortality among replicates, and misleading results from a lack of true replication. The results obtained from the two validation experiments conducted here indicated that the system was successfully designed, and the biotic and abiotic conditions were reproducible between our independent replicates.

Turner and Renegar (2017) suggested that oil spills near coral reefs are the only opportunities to assess the effects of acute oil contamination on corals in situ. To the best of our knowledge, no open/contained system has been constructed for studies on the effect of oil spills and chemical strategies used for remediation described in the literature. Most studies are performed in closed‐system microcosms due to operational and logistical difficulties. However, closed systems do not incorporate actual environmental factors to allow for reliable predictions of field situations (Reilly, 1999). According to Evans et al. (2010), approaches that integrate different methods on a larger scale could approximate these realistic levels. The use of mesocosm systems allows more precise modeling of several scenarios, in this case the exposure of coral to petroleum compounds and their impact on coral.

When possible, open systems are the best options for marine studies because they emulate a flow of nutrients that is closer to natural levels. Open systems also avoid the accumulation of metabolic products from the organisms under study. Mucus secreted by corals during the day can cover up to 95% of the surface of some coral species (Bessell‐Browne, Fisher, Duckworth, & Jones, 2017) and reach the equivalent of 30%–40% of their photosynthetic production (Crossland, Barnes, & Borowitzka, 1980) due to the high concentration of polysaccharides, lipids, and monosaccharides in coral mucus (Ducklow & Mitchell, 1979; Mitchell & Chet, 1975). Glasl, Herndl, and Frade (2016) showed that the mucus‐associated prokaryotic community, which is influenced by natural mucus shedding, affects coral health. Thus, when using either open or closed systems, it is important to have a low water residence time in aquariums to maintain realistic experimental conditions (Schindler, 1998), which could be affected by the increase of DOC from excess mucus. Some authors have observed that an increase in DOC can indirectly influence corals by causing side effects ranging from tissue recession to death (Haas et al., 2010; Kuntz, Kline, Sandin, & Rohwer, 2005). The higher DOC content in highly contaminated samples may be due to the possible increase of mucus production by stressed corals. DOC is rich in carbon sources, acting as a culture medium for heterotrophic microbes (Brown & Bythell, 2005). The increase of this heterotrophic community can lead to hypoxia and the death of coral (Smith et al., 2006). According to Meyer et al. (2016), this problem may also cause shifts in the microbial communities associated with corals and may reduce the rates of calcification and photosynthesis. The low DOC concentrations detected in the control samples in trials 1 and 2 suggest that the PROCORAIS mesocosm dealt effectively with the accumulation of mucus.

For studies of oil spills on coral reefs, a flow‐through system is more realistic than static exposure in a closed system, which is another advantage of the PROCORAIS Mesocosm. Cohen, Nissenbaum, and Eisler (1977) observed that mortality of corals occurred in oil‐exposure experiments with static exposure but that no mortality was observed in a flow‐through experiment. The faster mortality of corals in closed systems can be explained by the damage caused by the contaminant impact combined with the increase of other harmful compounds, such as DOC, that may not reflect realistic rates observed in nature (Haas et al., 2010).

The use of gravity to feed the mesocosm was a major advantage of this system and provided several important benefits, including reduced maintenance and risk due to pump failure. Among the major constraints for the dilution of chemical contaminants (e.g., oil) in mesocosm studies is the use of peristaltic pumps. Chemical contaminants may corrode or clog hoses and thus interfere with the functionality of the mesocosm. In addition, the electrical energy consumption of peristaltic pumps is high and not considered a sustainable practice. For this reason, the PROCORAIS Mesocosm was developed to use gravity rather than peristaltic pumps. The design used gravity‐fed flow that makes it unlikely to suffer equipment failures and was resistant to clogging that could lessen the reliability of results. Finally, the use of gravity feed from the 300‐L tanks provided a buffer during which the mesocosm could continue to function several hours in the case of pump failure.

In addition to being flexible and adjustable to numerous experimental configurations, this system can be used to study the effects of other chemical agents or climate change on marine organisms, as well as the efficacy of remediation approaches, such as marine probiotics. The present experimental results suggest that the mesocosm met the requirements of realism, adaptability, and number of replications needed for this type of experiment. As an example, Rosado et al. (2018) successfully performed an experiment in the PROCORAIS Mesocosm that was adapted to study corals stressed by increasing temperatures and challenged with the inoculation of the thermal‐dependent pathogen *Vibrio coralliilyticus*BAA‐450 on the coral *Pocillopora damicornis*. In this case, a beneficial microorganism consortium (BMC) for corals (Peixoto, Rosado, Leite, Rosado, & Bourne, 2017) was also tested against the pathogen challenge and thermal stress (Rosado et al., 2018). For this experiment, the flow system was closed in aquariums, with each aquarium having its own 26‐L tank to form a circulating flow, to avoid contamination of the environment by the pathogen and BMC inoculation.

When designing the PROCORAIS Mesocosm, it was critical to use materials that would not introduce contaminants that might influence the experiments while also considering the cost of such materials. Initially, Teflon was thought to be suitable for this mesocosm, because it is chemically inert, resistant to corrosion, and capable of repelling water and other chemical solvents with a low coefficient of friction (Rae & Brown, 2005; Renfrew & Lewis, 1946). However, we discovered that the use of Teflon would be cost‐prohibitive for such a complex system. Therefore, we chose the use of polypropylene tanks as a chemically resistant and cheaper material, and polyethylene and silicone hoses, as they are inert and do not interact chemically with other compounds (Rossmann, 1956; Shit & Shah, 2013). According to Kline et al. (2006), the long‐term use of plastic materials (petroleum derivatives) may alter the data of some chemicals in chronic contact in the presence of corrosion. These materials, when corroded, may also cause changes in the microorganisms associated with corals, leading to an imbalance of this symbiotic association, due to the formation of biofilm in these materials. However, due to the short‐term nature of our experiments, materials made of silicone, polyethylene, and polypropylene were effective in both trials, with no problems of corrosion, clogging, or risk of contamination.

The analyses performed to validate the PROCORAIS Mesocosm found the physical and chemical parameters to be generally stable and demonstrated the ability of the mesocosm to control environmental conditions, even among several experimental treatments. The salinity range was similar between treatments in the first trial and within the natural salinity range in seawater (33–35 g/L). In the second trial—when dilution was not adopted—mean salinity increased in all treatments. This behavior can be ascribed to the combined effects of evaporation and lack of seawater reposition, which lead to the higher salt concentration in the system. However, the salinity levels are near those at the beaches of Armação dos Búzios, Rio de Janeiro, which average 33–35 g/L and reach 37 g/L in cold temperatures. pH was within the natural range observed at the coral collection site (pH = 8–9).

High‐nutrient concentrations can have negative impacts on corals, including shifts from net accretion to net erosion of coral structures (Silbiger et al., 2018) and coral bleaching when coupled with thermal stress (Wang et al., 2018; Wiedenmann et al., 2013). The mesocosm maintained key water chemistry parameters (nitrite, nitrate, and ammonia) consistent throughout the experiment, with some exceptions during trial 2. However, increased ammonia concentrations over time in the closed system of trial 2 were consistent across treatments. Even with this increase in ammonia concentrations during trial 2, these values were still within the normal range of natural conditions according to the Brazilian CONAMA Resolution 357 (CONAMA, 2005; maximum value of nitrite = 200 µg/L and maximum values of nitrate and ammonia = 700 µg/L). These levels did not affect any other parameters, including coral health. The nutrient balance was maintained during the two trials, as were the temperature and DOC concentrations, which was important to avoid negative impacts due to high concentrations of these factors. The TPH and PAH values that were below detection limits in trial 1 were likely due to the oil concentration that was used (0.07%) and the fact that oil has very low solubility in water. In addition, the mesocosm setup with higher flow through likely caused an instantaneous dilution of the WSF. In contrast, the concentration of trial 2 (1%) emulates a worst‐case, high‐magnitude oil spill. According to an oil spill models developed by Applied Science Associates, Inc. (ASA, 2003) with Marlim oil, 2% of spilled oil would be present in water column after 12–13 days (when oil would reach coastal waters) under a worst‐case, high‐magnitude spill scenario (305,443 m^3^ of spilled oil). In this case, hydrocarbon pseudoconcentration would start at 6,500 mg/L (0.65%) and decrease to 1,000 mg/L (0.1%) within 30 days. This example shows that the level of oil concentration adopted in trail 2 was still higher than those identified with this model. The model also predicted that the weathering processes and the poor solubility of Marlim oil in water would favor low contaminant concentrations in the water column; conditions reproduced by the PROCORAIS Mesocosm.

Critical to the purpose of the PROCORAIS Mesocosm, we were able to detect impacts oil contamination on coral health. Specifically, higher oil concentrations (1%) had a negative impact on the symbiotic photosynthetic algae of *M. alcicornis*(i.e., *F_v_/F_m_)* after day 4 of the experiment. These results agree with those of DeLeo et al. (2015), who showed a decline in coral physiology through short‐term toxicological assays (0–96 hr) with a mixture of hydrocarbons. Additionally, in the PROCORAIS Mesocosm, when the concentration of oil in the tanks (“open ocean”) was increased by 14 times from the first to the second experiment, the WSFs of the oil were detectable in the “reefs” (aquariums).

In this study, we demonstrated the efficacy of the PROCORAIS Mesocosm to simulate oil spills of various magnitudes. This system presents several additional functionalities that are unique within the literature. First, the boxes holding the oil and water solution are exposed to realistic conditions of insolation and weathering. Thus, the mesocosm was able to simulate an oil spill and, very importantly, the time course of weathering until it reaches the reef. Second, we used natural sunlight with a light attenuating cloth that was able to mimic different depths in the reef while maintaining the characteristics of the light spectrum, which favors studies of PAM fluorimetry. Third, this system provides the ability to test various contaminants in combination with different temperatures to simulate the combined effects of climate change and anthropogenic stressors. Finally, a large part of the oil industry is located in developing countries where local universities do not have the financial resources to build expensive systems compared with developed countries. This system proved to be both effective and economical. Overall, this mesocosm system represents a unique structure and an opportunity to reproduce oil spills and their effects to coral reefs as realistically as possible, through the utilization of an open‐flow system that simulates the oil's route from open‐ocean areas to the reef, with true independent replicates. According to Sagarin et al. (2016), it is very difficult to obtain true replication in large‐scale mesocosms and to combine replication with inferences from complex systems, partly because a large mesocosm is very laborious to maintain. The PROCORAIS Mesocosm allows up to 4 true replications per treatment for a total of 13 treatments, or more replicates of fewer treatments with options to run with open or closed experiments.

Control of the abiotic factors and the biotic responses based on the treatments applied is critical for mesocosm experiments (Duarte et al., 2016; Richmond, 1993). The present study showed that the experiments performed with the PROCORAIS Mesocosm achieved this control even in different configurations. This flexibility is made possible by the use of the open‐ and closed‐flow designs and the use of materials that are nonreactive with a range of chemical additives for experiments. Although the mesocosm system was developed in this case to study the effects of oil spills on coral reefs, it is clear that it could help other researchers to develop their own mesocosms and improve upon it from our findings and insights.

## CONFLICT OF INTEREST

The authors declare no competing financial interests.

## AUTHOR CONTRIBUTIONS

Duarte, G.; Rosado, A.S.; Soriano, A.; and Peixoto, R.S. involved in mesocosm conception and design; Duarte, G.; Villela, H.; Silva, D.; Rosado, A.S.; Soriano, A.; and Peixoto, R.S. involved in experimental design; Duarte, G.; Silva, D.; Rosado, P.; Ferreira, E.; Santos, H.; Villela, H.; and Peixoto, R.S involved in mesocosm setup and adjustments; Silva, D.; Villela, H.; Rosado, P., Duarte, G., Soriano, A.; Rosado, J.G.; Santos, H.; and Peixoto, R.S. involved in acquisition of data (conducting of the experiments).Silva, D.; Duarte, G.; Villela, H.; Soriano, A.; and Peixoto, R.S. involved in analyses and interpretation of the data. Silva, D.; Duarte, G.; and Peixoto, R.S. drafted the manuscript. All authors involved in critical revision. Peixoto, R.S. provided financial support.

## Data Availability

Data supporting this manuscript will be made available on ResearchGate following acceptance.
